# FasTag: Automatic text classification of unstructured medical narratives

**DOI:** 10.1371/journal.pone.0234647

**Published:** 2020-06-22

**Authors:** Guhan Ram Venkataraman, Arturo Lopez Pineda, Oliver J. Bear Don’t Walk IV, Ashley M. Zehnder, Sandeep Ayyar, Rodney L. Page, Carlos D. Bustamante, Manuel A. Rivas

**Affiliations:** 1 Department of Biomedical Data Science, School of Medicine, Stanford University, Stanford, CA, United States of America; 2 Department of Biomedical Informatics, Vagelos College of Physicians and Surgeons, Columbia University, New York, NY, United States of America; 3 Fauna Bio, San Francisco, CA, United States of America; 4 Department of Clinical Sciences, College of Veterinary Medicine and Biomedical Sciences, Colorado State University, Fort Collins, CO, United States of America; 5 Chan Zuckerberg Biohub, San Francisco, CA, United States of America; University of Lincoln, UNITED KINGDOM

## Abstract

Unstructured clinical narratives are continuously being recorded as part of delivery of care in electronic health records, and dedicated tagging staff spend considerable effort manually assigning clinical codes for billing purposes. Despite these efforts, however, label availability and accuracy are both suboptimal. In this retrospective study, we aimed to automate the assignment of top-level International Classification of Diseases version 9 (ICD-9) codes to clinical records from human and veterinary data stores using minimal manual labor and feature curation. Automating top-level annotations could in turn enable rapid cohort identification, especially in a veterinary setting. To this end, we trained long short-term memory (LSTM) recurrent neural networks (RNNs) on 52,722 human and 89,591 veterinary records. We investigated the accuracy of both separate-domain and combined-domain models and probed model portability. We established relevant baseline classification performances by training Decision Trees (DT) and Random Forests (RF). We also investigated whether transforming the data using MetaMap Lite, a clinical natural language processing tool, affected classification performance. We showed that the LSTM-RNNs accurately classify veterinary and human text narratives into top-level categories with an average weighted macro F1 score of 0.74 and 0.68 respectively. In the “neoplasia” category, the model trained on veterinary data had a high validation accuracy in veterinary data and moderate accuracy in human data, with F1 scores of 0.91 and 0.70 respectively. Our LSTM method scored slightly higher than that of the DT and RF models. The use of LSTM-RNN models represents a scalable structure that could prove useful in cohort identification for comparative oncology studies. Digitization of human and veterinary health information will continue to be a reality, particularly in the form of unstructured narratives. Our approach is a step forward for these two domains to learn from and inform one another.

## Introduction

### Motivation

The increasingly worldwide adoption of electronic health records (EHRs) has populated clinical databases with millions of clinical narratives (descriptions of actual clinical practice). However, given the nature of the medical enterprise, a big portion of the data being recorded is in the form of unstructured free-text clinical notes. Cohorts of individuals with similar clinical characteristics require quality phenotype labels, oftentimes not readily available alongside clinical notes, to be studied adequately.

In place of such labeling, diagnostic codes are the most common surrogates to true phenotypes. In routine clinical practice, dedicated tagging staff read clinical narratives and assign these diagnostic codes to patients’ diagnoses from one or both of two coding systems: the International Classification of Diseases (ICD) and the Systematized Nomenclature of Medicine (SNOMED) [1]. However, this time-consuming, error-prone task leads to only 60–80% of the assigned codes reflecting actual patient diagnoses [2], misjudgment of severity of conditions, and/or omission of codes altogether. The relative inaccuracy of oncological medical coding [3–6] affects the quality of cancer registries [7] and cancer prevalence calculations [8–10], for example. Poorly-defined disease types and poorly-trained coding staff who overuse the “not otherwise specified” code when classifying text exacerbate the problem.

Challenges in clinical coding also exist in veterinary medicine in the United States, where neither clinicians nor medical coders regularly apply diagnosis codes to veterinary visits. There are few incentives for veterinary clinicians to annotate their records; a lack of 1) a substantial veterinary third-party payer system and 2) legislation enforcing higher standards of veterinary EHRs (the U.S. Health Information Technology for Economic and Clinical Health Act of 2009 sets standards for human EHRs) compound the problem. Billing codes are thus rarely applicable across veterinary institutions unless hospitals share the same management structure and records system; even then, hospital-specific modifications exist. Less than five academic veterinary centers of a total of thirty veterinary schools in the United States have dedicated medical coding staff to annotate records using SNOMED-CT-Vet [11], a veterinary extension of SNOMED constructed by the American Animal Hospital Association (AAHA) and maintained by the Veterinary Terminology Services Laboratory at the Virginia-Maryland Regional College of Veterinary Medicine [12].

The vast majority of veterinary clinical data is stored as free-text fields with very low rates of formal data curation, making data characterization a tall order. Further increasing variance in the data, veterinary patients come from many different environments, including hospitals [13], practices [14], zoos [15], wildlife reserves [16], army facilities [17], research facilities [18], breeders, dealers, exhibitors [19], livestock farms, and ranches [20].

It is thus important that a general method, agnostic of patient environment, is able to categorize EHRs for cohort identification solely based on free-text.

### A primer on automatic text classification

Automatic text classification is an emerging field that uses a combination of tools such as human medical coding, rule-based systems queries [21], natural language processing (NLP), statistical analyses, data mining, and machine learning (ML) [22]. In a previous study [23], we have shown the feasibility of automatic annotation of veterinary clinical narratives across a broad range of diagnoses with minimal preprocessing, but further exploration is needed to probe what we can learn from human-veterinary comparisons. Automatically adding meaningful disease-related tags to human and veterinary clinical notes using the same machinery would be a huge step forward in that exploration and could facilitate cross-species findings downstream.

Said integration has the potential to improve both veterinary and human coding accuracy as well as comparative analyses across species. Comparative oncology, for example, has accelerated the development of novel human anti-cancer therapies through the study of companion animals [24], especially dogs [25–28]. The National Institute of Health recently funded a multi-center institution called the Arizona Cancer Evolution Center (ACE) that aims to integrate data from a broad array of species to understand the evolutionarily conserved basis for oncology. As this group utilizes animal clinical and pathology data to identify helpful traits like species-specific cancer resistance, they would greatly benefit from improved cohort discovery through automated record tagging.

15 out of 30 veterinary schools across the United States have formed partnerships with their respective medical schools in order to perform cross-species translational research within the Clinical and Translational Science Award One Health Alliance (COHA, [29]). Of these schools, only two have active programs to assign disease codes to their medical records. The data for the rest represents the very use case of automatic text classification.

Automatic medical text classification aims to reduce the human burden of handling unstructured clinical narratives. These computational NLP methods can be divided into two groups: a) semantic processing and subsequent ML; and b) deep learning.

#### Semantic processing and subsequent ML

Semantic processing methods range from simple dictionary-based keyword-matching techniques and/or direct database queries to tools capable of interpreting the semantics of human language through lemmatization (removal of inflectional word endings), part-of-speech tagging, parsing, sentence breaking, word segmentation, and entity recognition [30]. Building the underlying dictionaries and manually crafting the rules that capture these diverse lexical elements both require time and domain expertise.

There is a growing interest in medical concept classification for clinical text; as such, many domain-specific semantic NLP tools (with various objectives, frameworks, licensing conditions, source code availabilities, language supports, and learning curves) have been developed for the medical setting. Such tools include MedLEE [31], MPLUS [32], MetaMap [33], KMCI [34], SPIN [35], HITEX [36], MCVS [37], ONYX [38], MedEx [39], cTAKES [40], pyConTextNLP [41], Topaz [42], TextHunter [43], NOBLE [44], and CLAMP [45]. However, there is no single NLP tool that can handle the broad problem of general medical concept classification. Instead, each method solves specific problems and applies its unique set of constraints.

After clinical narratives are fed into the above semantic NLP tools, various clinical “concepts” or “terms” (e.g. conditions, diseases, age, body parts, periods of time, etc.) are extracted. These concepts can then be represented in a “term-document matrix,” which shows frequencies of the terms across documents. These frequencies can be used raw as features in a ML model, but more often than not, choices are made to transform these features to more meaningful spaces. This can be done via term frequency-inverse document frequency (tf-idf, which assigns weights to terms based on the frequency of the term in both the document of interest as well as the corpus at large), other vectorization techniques like Word2Vec [46], or manually curated rules.

Predictive ML models (like Decision Trees [DTs], Random Forests [RFs], and Support Vector Machines [SVMs] [47]) that operate on the raw or transformed term-document matrix use this training data (input features and “ground-truth” labels) to make accurate predictions or decisions on unseen test data without explicit instructions on how to do so. They have been shown to achieve high classification accuracy in human [48, 49] and veterinary [50] free-text narratives for diseases well-represented in training datasets (e.g. diabetes, influenza, and diarrhea). However, these models generally do not classify under-represented diseases or conditions well, and opportunities for both public data generation and methodological innovation lie in this space [50].

#### Deep learning

Deep learning (DL) methods eliminate the need of feature engineering, harmonization, or rule creation. They learn hierarchical feature representations from raw data in an end-to-end fashion, requiring significantly less domain expertise than traditional ML approaches [51].

DL is quickly emerging in the literature as a viable alternative method to traditional ML for the classification of clinical narratives [47]. The technique can help in the recognition of a limited number of categories from biomedical text [52, 53]; identify psychiatric conditions of patients based on short clinical histories [54]; and accurately classify whether or not radiology reports indicate pulmonary embolism [55, 56] whilst outperforming baseline non-DL-based methods (e.g. RFs or DTs). Previous studies have shown the possibility of using DL to label clinical narratives with medical subspecialties [57] (e.g. cardiology or neurology) or medical conditions [58] (e.g. advanced cancer or chronic pain), outperforming concept-extraction based methods. Furthermore, the use of DL to analyze clinical narratives has also facilitated the prediction of relevant patient attributes, such as in-hospital mortality, 30-day unplanned readmission, prolonged length of stay, and final discharge diagnosis [59].

Traditional NLP methods boast interpretability and flexibility but come at the steep cost of data quality control, formatting, normalization, domain knowledge, and time needed to generate meaningful heuristics (which oftentimes are not even generalizable to other datasets). Automatic text classification using DL is thus a logical choice to bypass these steps, classifying medical narratives from EHRs by solely leveraging big data. We expect that our efforts could facilitate rapid triaging of documents and cohort identification for biosurveillance.

## Materials and methods

### Ethics statement

This research was reviewed and approved by Stanford’s Institutional Review Board (IRB), which provided a non-human subject determination under eProtocol 46979. Consent was not required.

### Study design

This retrospective cross-sectional chart review study uses medical records collected routinely as part of clinical care from two clinical settings: the veterinary teaching hospital at Colorado State University (CSU) and the Medical Information Mart for Intensive Care (MIMIC-III) from the Beth Israel Deaconess Medical Center in Boston, Massachusetts [60]. Both datasets were divided in two smaller datasets—training datasets containing 70% of the original datasets (used to build TensorFlow [61] DL models), and validation datasets containing 30% of the original datasets.

The goal of our model was to predict top-level ICD version 9 (ICD-9) codes, which we considered our “ground-truth” labels. These codes are organized in a hierarchical fashion, with the top levels representing the grossest possible descriptors of clinical diseases or conditions (e.g., “neoplasia”). The MIMIC-III dataset provides ICD-9 codes for all its patients as-is. However, veterinary codes from the CSU were coded using SNOMED-CT, and thus needed to be converted to their closest equivalent top-level ICD-9 codes. Mapping between SNOMED-CT and ICD-9 codes was a challenging task but promoted semantic interoperability between our two domains. Table 1 shows our mapping between ICD (versions 9 and 10) codes and their counterparts in SNOMED-CT (including the Veterinary extension, SNOMED-CT-Vet). This mapping, which then allowed us to generate “ground-truth” labels for the veterinary data,was manually curated by two board-certified veterinarians trained in clinical coding (co-authors AZM and RLP). We also wanted to investigate the effect of using MetaMap [33], a NLP tool that extracts clinically-relevant terms, on our clinical narratives. Specifically, we explored whether or not training our models on these “MetaMapped” free-texts (that only contain extracted UMLS terms) would improve accuracy.

**Table 1:** Top-level coding mapping between ICD-9, ICD-10, and SNOMED-CT.

Top-level category	Description	ICD-9	ICD-10	SNOMED-CT
1	Infectious and parasitic diseases	001-139	A00-B99	105714009, 68843000, 78885002, 344431000009103, 338591000009108, 40733004, 17322007
2	Neoplasms	140-239	C00-D49	723976005, 399981008
3	Endocrine, nutritional and metabolic diseases, and immunity disorders	240-279	E00-E90	85828009, 414029004, 473010000, 75934005, 363246002, 2492009, 414916001, 363247006, 420134006, 362969004
4	Diseases of blood and blood-forming organs	280-289	D50-D89	271737000, 414022008, 414026006, 362970003, 11888009, 212373009, 262938004, 405538007
5	Mental disorders	290-319	F00-F99	74732009
6	Diseases of the nervous system	320-359	G00-G99	118940003, 313891000009106
7	Diseases of sense organs	360-389	H00-H59, H60-H95	50611000119105, 87118001, 362966006, 128127008, 85972008
8	Diseases of the circulatory system	390-459	I00-I99	49601007
9	Diseases of the respiratory system	460-519	J00-J99	50043002
10	Diseases of the digestive system	520-579	K00-K93	370514003, 422400008, 53619000
11	Diseases of the genitourinary system	580-629	N00-N99	42030000
12	Complications of pregnancy, childbirth, and the puerperium	630-679	O00-O99	362972006, 173300003, 362973001
13	Diseases of the skin and subcutaneous tissue	680-709	L00-L99	404177007, 414032001, 128598002
14	Diseases of the musculoskeletal system and connective tissue	710-739	M00-M99	105969002, 928000
15	Congenital anomalies	740-759	Q00-Q99	111941005, 32895009, 66091009
16	Certain conditions originating in the perinatal period	760-779	P00-P96	414025005
17	Injury and poisoning	800-899	S00-T98	85983004, 75478009, 77434001, 417163006

Mapping of top-level categories was manually curated by two board-certified veterinarians trained in clinical coding.

**Fig 1:**
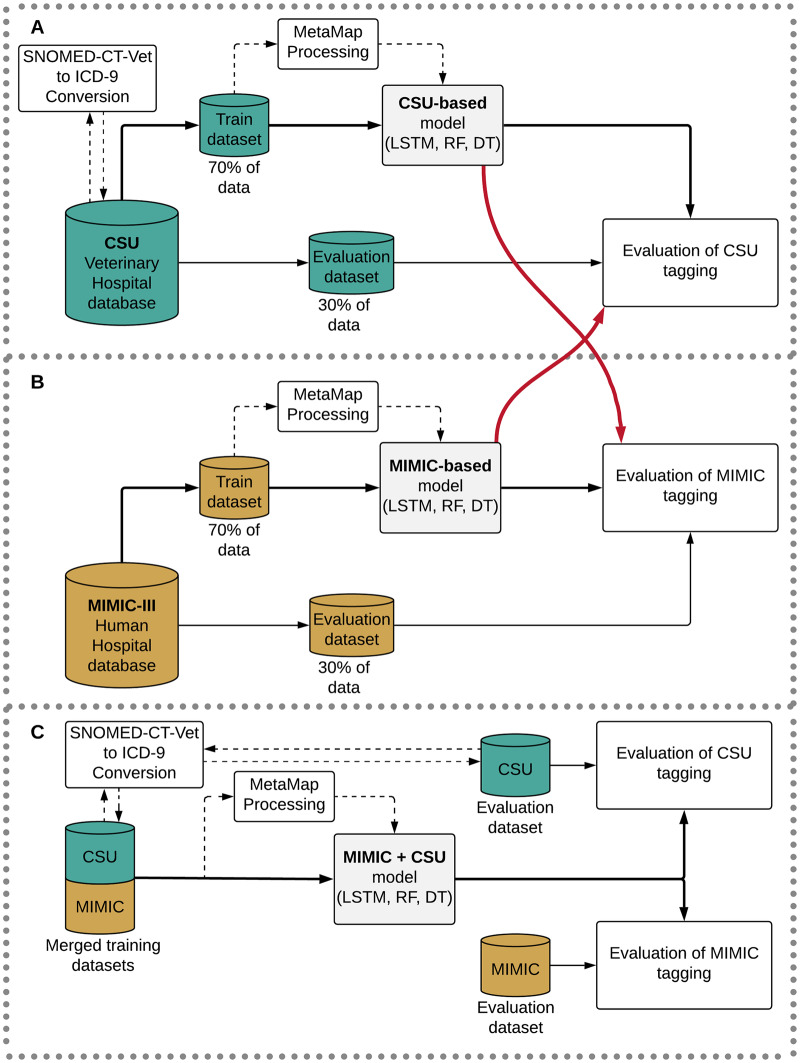
Diagram of the training and evaluation design. Relevant acronyms: MIMIC: Medical Information Mart for Intensive Care; CSU: Colorado State University; MetaMap, a tool for recognizing medical concepts in text; LSTM: long-short term memory recurrent neural network classifier; RF: Random Forest classifier; DT: Decision Tree classifier.

Finally, we measured the validation accuracy of the models, calculating the F1 score of our model and relevant non-DL baselines in each top-level disease category. We also explored the possibility of out-of-domain generalization, testing the MIMIC-trained model on the CSU validation data and vice versa (and ran separate tests for “MetaMapped” versions of each dataset, as well). Finally, we investigated the effect of merging the MIMIC and CSU training datasets to test the efficacy of data augmentation. Fig 1 shows a diagram of our study design. Our code to run all models can be found in a public repository (https://github.com/rivas-lab/FasTag).

### Data

#### Veterinary medical hospital at Colorado State University (CSU)

The CSU is a tertiary care referral teaching hospital with inpatient and outpatient facilities, serving all specialties of veterinary medicine. After consultation, veterinarians enter patient information into a custom-built veterinary EHR, including structured fields such as entry and discharge dates, patient signalment (species, breed, age, sex, etc.), and SNOMED-CT-Vet codes. There are also options to input free-text clinical narratives with various sections including history, assessment, diagnosis, prognosis, and medications. These records are subsequently coded; the final diagnostic codes represent single or multiple specific diagnoses or post-coordinated expressions (a combination of two or more concepts). For our study, we used the free-text clinical narratives (data input) and the SNOMED-CT-Vet codes (labels), which we subsequently converted to ICD-9 top-level codes as per Table 1.

#### Medical Information Mart for Intensive Care (MIMIC-III)

The Beth Israel Deaconess Medical Center is a tertiary care teaching hospital at Harvard Medical School in Boston, Massachusetts. The MIMIC-III database, a publicly available dataset which we utilize in this study, contains information on patients admitted to the critical care unit at the hospital [60]. These records are coded for billing purposes and have complete diagnoses per patient (the database is publicly available, and thus represents the best possible medical coding annotation scenario for a hospital). We were interested in the free-text hospital discharge summaries (data input) and the corresponding ICD-9 codes (labels) for the patients in this database. Free-text fields in MIMIC-III have been de-identified to protect privacy.

#### Comparing the sources

The CSU dataset contains medical records from 33,124 patients and 89,591 hospital visits between February 2007 and July 2017. Patients encompassed seven mammalian species, including dogs (*Canis Lupus*, 80.8%), cats (*Felis Silvestris*, 11.4%), horses (*Equus Caballus*, 6.5%), cattle (*Bos Taurus*, 0.7%), pigs (*Sus Scrofa*, 0.3%), goats (*Capra hircus*, 0.2%), sheep (*Ovis Aries*, 0.1%), and other unspecified mammals (0.1%). In contrast, the MIMIC-III database contains medical records from 38,597 distinct human adult patients (aged 16 years or above) and 7,870 neonates admitted between 2001 and 2008, encompassing 52,722 unique hospital admissions to the critical care unit between 2001 and 2012. Table 2 summarizes the category breakdowns of both databases. For this analysis, only those patients with a diagnosis in their record were considered.

**Table 2:** Database statistics of patients, records, and species (records with diagnosis).

	CSU	MIMIC
**Data**		
Medical Records	89,591	52,722
Patients	33,124	41,126
Hospital Visits	89,591	49,785
**Species**		
Humans (Homo Sapiens)	n.a.	52,722
Dogs (Canis Lupus)	72,420	n.a.
Cats (Felis Silvestris)	10,205	n.a.
Horses (Equus Caballus)	5,819	n.a.
Other mammals	1,147	n.a.
**Category**		
Infectious	11,454	10,074
Neoplasia	36,108	6,223
Endo-Immune	17,295	24,762
Blood	10,171	13,481
Mental	511	10,989
Nervous	7,488	9,168
Sense organs	15,085	2,688
Circulatory	8,733	30,054
Respiratory	11,322	17,667
Digestive	22,776	14,646
Genitourinary	8,892	14,932
Pregnancy	136	133
Skin	21,147	4,241
Musculoskeletal	22,921	6,739
Congenital	3,347	2,334
Perinatal	54	3,661
Injury	9,873	16,121

The mappings in Table 1 were used to generate the categories and numbers presented here in Table 2. The seventeen categories represent the text classification labels.

### MetaMap

Our hypothesis was that there would be a plethora of extraneous information, domain- and setting-specific misspellings, abbreviations, and jargon in the clinical narratives in both human and veterinary settings. In order to potentially resolve some of these issues, we used MetaMap Lite [62], a NLP tool which leverages the Unified Medical Language System (UMLS) Metathesaurus to identify SNOMED [63] or ICD-9 [64] codes from clinical narratives. MetaMap’s algorithm includes five steps: 1) parsing of text into simple noun phrases; 2) variant generation of phrases to include all derivations of words (i.e. synonyms, acronyms, meaningful spelling variants, combinations, etc.); 3) candidate retrieval of all UMLS strings that contains at least one variant from the previous step; 4) evaluation and ranking of each candidate, mapping between matched term and the Metathesaurus concept using metrics of centrality, variation, coverage, and cohesiveness; 5) construction of complete mappings to include those mappings that are involved in disjointed parts of the phrase (e.g. ‘ocular’ and ‘complication’ can together be mapped to a single term, ‘ocular complication’). MetaMap incorporates the use of ConText [65], an algorithm for the identification of negation in clinical narratives.

We proceeded to make a “MetaMapped” version of each training and validation dataset to see whether extracting and inputting only clinical terms from the narratives into the models would increase their accuracy. For additional information and examples of how we used and evaluated MetaMap, please refer to S1 Text and S1–S3 Tables.

### Model architecture

We chose a long short-term memory (LSTM) recurrent neural network (RNN) architecture (which is able to handle variable-length sequences while using previous inputs to inform current time steps) for this multi-label text classification task [66]. The LSTM shares parameters across time steps as it unrolls, which allows it to handle sequences of variable length. In this case, these sequences are a series of word “embeddings” (created by mapping specific words to corresponding numeric vectors) from clinical narratives. Words are represented densely (rather than sparsely, as in Bag-of-Words or tf-idf models) using the Global Vectors for Word Representation (GloVe) [67] word embeddings. These embeddings learn a vector space representation of words such that words with similar contexts appear in a similar vector space and also capture global statistical features of the training corpus.

LSTMs have proven to be flexible enough to be used in many different tasks, such as machine translation, image captioning, medication prescription, and forecasting disease diagnosis using structured data [66]. The RNN can efficiently capture sequential information and theoretically model long-range dependencies, but empirical evidence has shown this is difficult to do in practice [68]. The sequential nature of text lends itself well to LSTMs, which have memory cells that can maintain information for over multiple time steps (words) and consist of a set of gates that control when information enters and exits memory, making them an ideal candidate architecture.

We first trained FasTag, the LSTM model, over a variety of hyperparameters on MIMIC data and calculated the model’s validation accuracy over all combinations of them, finding the set of [learning rate = 0.001, dropout rate = 0.5, batch size = 256, training epochs = 100, hidden layer size = 200, LSTM layers = 1] to be the optimal setting. We proceeded to use this hyperparameter set for all FasTag models trained, assuming that this set would be amenable to the task at hand regardless of training dataset. We then proceeded to train a set of six models on three datasets (MIMIC, CSU, and MIMIC+CSU) where each dataset had a version that was processed with MetaMap and a version that was not.

### Evaluation

We aimed to characterize the performance of FasTag in both absolute and relative senses by establishing its empirical classification accuracy and the accuracy of non-DL alternatives. Several ML classifiers have similarly aimed to classify clinical narratives [47, 69]. We selected two of these classifiers (DTs and RFs) as relevant non-DL baseline comparator methods. DTs are ML models constructed around a branching boolean logic [70]. Each node in the tree can take a decision that leads to other nodes in a tree structure; there are no cycles allowed. The RF classifier is an ensemble of multiple DTs created by randomly selecting samples of the training data. The final prediction is done via a consensus voting mechanism of the trees in the forest.

We featurized the narratives using tf-idf, a statistic that reflects word importance in the context of other documents in a corpus and a standard ML modeling strategy for representing text, to convert the narratives into a text-document matrix [47]. The hyperparameters of both baseline models (DT and RF), like for FasTag, were tuned on the validation set.

For all models we trained (FasTag, DT, and RF), we used the same validation set evaluation metrics previously reported for MetaMap [62]: a) precision, defined as the proportion of documents which were assigned the correct category; b) recall, defined as the proportion of documents from a given category that were correctly identified; and c) F1 score, defined as the harmonic average of precision and recall. Formulas for these metrics are provided below:

$$\begin{array}{c} \text{Precision}=\frac{\text{True}\;\text{Positive}}{\text{True}\;\text{Positive}+\text{False}\;\text{Positive}} \end{array}$$ (1)

$$\begin{array}{c} \text{Recall}=\frac{\text{True}\;\text{Positive}}{\text{True}\;\text{Positive}+\text{False}\;\text{Negative}} \end{array}$$ (2)

$$\begin{array}{c} F_{1}=2*\frac{\text{Precision}*\text{Recall}}{\text{Precision}+\text{Recall}} \end{array}$$ (3)

Our task is framed as a multi-label classification problem, where each approach predicts multiple top-level ICD-9 categories for each observation using a single model. In order to combine all class-specific F1 scores, we averaged the F1 score for each label, weighting the labels by their supports (the number of true instances for each label, to account for label imbalance).

#### Domain adaptation

The portability of trained algorithms on independent domains has previously been used as a metric of model robustness in systems that leverage NLP and ML [71]. We evaluated the ability of our trained FasTag LSTM models to be used in a cross-species context. We utilized the MIMIC-trained model to classify the medical records in the CSU database and vice versa, assessing performance as before. We also assessed the classifier trained on the combined training set.

## Results

We investigated the application of FasTag to free-text unstructured clinical narratives on two cohorts: veterinary medical records from CSU, and human medical records in the MIMIC-III database.

We trained FasTag (as well as DT/RF baselines) on the human, veterinary, and merged (human and veterinary) datasets and tested each on their own domain as well as the other domains. We built FasTag using the Python programming language (version 2.7), TensorFlow [61] (version 1.9), and the scikit-learn library (version 0.19.2) [72]; we built the baselines using Python and scikit-learn as well. The training was performed on an Amazon^®^ Deep Learning AMI, a cloud-based platform running the Ubuntu operating system with pre-installed CUDA dependencies. FasTag’s training procedure was epoch-based; that is, our data was split into “batches” of size 256, and we calculated cross-entropy loss and updated the model using the Adam optimizer after each of these batches were input into the model. An “epoch” is said to have finished every time the entire dataset has passed through the model in batches. As is standard with most epoch-based model-training procedures, we trained FasTag until our validation loss increased between epochs three consecutive times. For the DT and RF baselines, we performed validation-set model selection across a grid of hyperparameters, including: information criterion; max features; max depth (of tree[s]); number of estimators; and tf-idf vector normalization type (L1 or L2). Average weighted macro F1 scores for models across all categories are shown in Table 3; a full list of F1 scores by category can be found in S4 Table. The “neoplasia” category results, which we found notable, are shown in Table 4.

**Table 3:** Average F_1_ scores using various training and validation dataset combinations for all categories.

Configuration			Model evaluation (Weighted F_1_ score)		
Training	Validation	MetaMap	DT	RF	LSTM
MIMIC	MIMIC	No	0.60	0.64	**0.65**
		Yes	0.60	0.63	**0.70**
CSU	CSU	No	0.55	0.61	**0.72**
		Yes	0.54	0.60	**0.75**
MIMIC	CSU	No	0.22	0.24	**0.28**
		Yes	0.23	0.20	**0.31**
CSU	MIMIC	No	**0.31**	0.20	0.23
		Yes	0.28	0.19	**0.36**
MIMIC + CSU	CSU	No	0.57	0.62	**0.67**
		Yes	0.57	0.62	**0.76**
MIMIC + CSU	MIMIC	No	0.60	**0.63**	0.58
		Yes	0.60	**0.63**	0.60
MIMIC + CSU	MIMIC + CSU	No	0.59	0.64	**0.68**
		Yes	0.59	0.63	**0.71**
**Average**			0.489	0.506	**0.571**

Evaluation metrics for Decision Tree (DT), Random Forest (RF), and the FasTag Long Short Term Memory (LSTM) Recurrent Neural Network on validation datasets with and without MetaMap term extraction. Bolded and underlined numbers represent the best scores for the specific configuration of training data, validation data, and MetaMap toggle.

**Table 4:** F_1_ scores using various training and validation dataset combinations for the “neoplasia” category.

Configuration			Model evaluation (Weighted F_1_ score)		
Training	Validation	MetaMap	DT	RF	LSTM
MIMIC	MIMIC	No	0.39	0.45	**0.66**
		Yes	0.4	0.45	**0.76**
CSU	CSU	No	0.81	0.86	**0.91**
		Yes	0.8	0.86	**0.91**
MIMIC	CSU	No	0.3	0.53	**0.69**
		Yes	0.45	0.37	**0.75**
CSU	MIMIC	No	0.46	0.58	**0.70**
		Yes	0.5	**0.58**	0.54
MIMIC + CSU	CSU	No	0.74	0.8	**0.87**
		Yes	0.74	0.8	**0.87**
MIMIC + CSU	MIMIC	No	0.4	0.47	**0.67**
		Yes	0.42	0.45	**0.72**
MIMIC + CSU	MIMIC + CSU	No	0.81	**0.86**	0.85
		Yes	0.81	0.86	**0.90**
**Average**			0.574	0.637	**0.771**

Evaluation metrics for the “neoplasia” category Decision Tree (DT), Random Forest (RF), and the FasTag Long Short Term Memory (LSTM) Recurrent Neural Network on validation datasets with and without MetaMap term extraction. Bolded and underlined numbers represent the best scores for the specific configuration of training data, validation data, and MetaMap toggle.

## Discussion

Applying DL to unstructured free-text clinical narratives in electronic health records offers a relatively simple, low-effort means to bypass the traditional bottlenecks in medical coding. Circumventing the need for data harmonization was very important for the datasets, which contained a plethora of domain- and setting-specific misspellings, abbreviations, and jargon (these issues would have greatly impacted the performance of standard ML models, and indeed, these were the cause of misclassifications by FasTag, as well). MetaMap was useful in this regard given its ability to parse clinical data, but much work is still needed to improve recognition of terms in veterinary and human domains (as evidenced by only low-to-moderate gains, and in some cases, losses, in performance in “MetaMapped” datasets [S4 Table]).

There is moderate evidence of domain adaptation (where a model trained on MIMIC data is useful in the CSU validation set, or vice versa) in the “neoplasia” category, with F1 scores of 0.69-0.70 (Table 4). This process involved training a model on the data in one database and testing on the data in the other without fine-tuning. It is evident that the high classification accuracy (F1 score = 0.91) obtained by the CSU model in the neoplasia category is decreased when testing the same model on the MIMIC data. One possible explanation is the difference in clinical settings; CSU is a tertiary care veterinary hospital specializing in oncological care, and the clinical narratives that arise in a critical care unit like in the MIMIC dataset do not necessarily compare. Moreover, the records were not coded in the same way, the clinicians did not receive the same training, and the documents apply to different species altogether (see S3 Table for an example of an example narrative unique to veterinary care). Despite these differences, however, our LSTM model was general enough to be able to accurately classify medical narratives at the top level of depth independently in both datasets. The achieved cross-domain accuracy is thus nonetheless encouraging. Given enough training data and similar-enough clinical narratives, one could conceivably imagine a general model that is highly effective across domains.

Models performed usually better on their respective validation datasets in those categories with more training samples. For example, the CSU-trained model (25,276 samples) had significantly better performance in the “neoplasia” category than the MIMIC-trained model (4,356 samples), while the MIMIC-trained model (21,038 samples) had better performance in the diseases of the circulatory system category than the CSU-trained model (6,133 samples). The corollary to this is that the biggest impediment to model performance within a category was the lack of training data. Unlike in the genetics community, where there exist hundreds of thousands of research samples available to researchers through DUAs [73], there is definitely a dearth of de-identified clinical text narratives alongside quality labels like in MIMIC-III. Along the same vein, when training on a mixed dataset of MIMIC and CSU data, we observed that the performance of the resultant classifier was significantly better on CSU than MIMIC validation data across various top-level categories (S4 Table). We hypothesize that a combination of the inherent differences in data across the two domains and the larger number of CSU records in the training set led to this performance gap. We additionally hypothesize that mixing training data from more similar data sources, in contrast, would result in strictly better performance outcomes on test data from both sources.

Insights gained through this work on generalizing across clinical and veterinary domains could be informative in training models attempting to generalize across different clinical institutions but within the same clinical domain. Linguistic variation within a clinical domain is due to factors like geography, clinical specialty, and patient population, among others. This variation manifests across many characteristics such as syntax [74–76], semantics [77], and workflow procedures [78]. The common practices to address domain heterogeneity are to re-train models from scratch [78] or to utilize domain adaptation techniques like distribution mapping [79] for the task of interest. Overall, we hypothesize that adapting models across clinical institutions will bear better results than when adapting them across clinical domains (like we have attempted in this work).

The usefulness of even top-level characterizations in the veterinary setting cannot be understated; usually, a veterinarian must read the full, unstructured text in order to get any information about the patient they are treating. Rapid selection of documents with specific types of clinical narratives (such as oncological cases, which our model performed well on) could lead to better cohort studies for comparative research. The repeated use of a series of such LSTM models for subsequent, increasingly-specific classifications thus represents a scalable, hierarchical tagging structure that could prove extremely useful in stratifying patients by specific diseases, severities, and protocols.

## Conclusion

In this era of increasing deployment of EHRs, it is important to provide tools that facilitate cohort identification. Our deep learning approach, FasTag, was able to automatically classify medical narratives with minimal human preprocessing. In a future with enough training data, it is possible to foresee a scenario in which these models can accurately tag every clinical concept, regardless of data input. The expansion of veterinary data availability and the subsequently enormous potential of domain adaptation like we saw in the neoplasia category could prove to be exciting chapters in reducing bottlenecks in public health research at large; it is thus of critical importance to continue studying novel sources of data that can rapidly be used to augment classification models.

A reliable addition to existing rule-based and natural language processing strategies, deep learning is a promising tool for accelerating public health research.

## Supporting information

S1 Text Evaluation of MetaMap on veterinary records. (DOCX)

S1 Table NLP (MetaMap) evaluation. Comparison of reviewer and MetaMap term extractions for 19 records. True Positive (TP) = MetaMap correctly matched term to code; False Negative (FN) = MetaMap did not extract a term the experts did; False Positive (FP) = MetaMap matched a term not identified by the expert; Total = Total number of terms matched in that document.
(XLSX)

S2 Table Confusion matrix for MetaMap Lite evaluation on veterinary data. (XLSX)

S3 Table Example of free-text and MetaMap-extracted veterinary record. A 2-year old female dog patient with recurrent otitis and allergic dermatitis. Both the narrative (left) and the “MetaMapped” version (right) show that the treatment included prednisone (among other important clinical details). For the purposes of this manuscript, the pet and owner’s name were manually de-identified.
(XLSX)

S4 Table Classification performance across categories and methods. Sheet 1: Long Short Term Memory (LSTM) Recurrent Neural Network (RNN) [FasTag]; Sheet 2: Decision Trees (DT); Sheet 3: Random Forests (RF).
(XLSX)

S1 Fig F1 scores by category using various training configurations. Training with CSU data (green), MIMIC data (yellow) or MIMIC+CSU (purple); validating on CSU data (Panel A) or MIMIC data (Panel B). The color of the category text is darkened (black) and the box made bigger if it surpasses the threshold of an F1 score of at least 0.70 (dotted horizontal line).
(TIF)
