# Supplementary material for: FasTag: Automatic text classification of unstructured medical narratives
Source: PLoS One. 2020 Jun 22;15(6):e0234647. doi: 10.1371/journal.pone.0234647 (PMC7307763; doi:10.1371/journal.pone.0234647)
Supplement: S1 Text — (DOCX) [file pone.0234647.s001.docx]

# **Evaluation of MetaMap on veterinary records**

MetaMap Lite has not previously been applied to veterinary data. As such, we endeavored to verify that the software works as expected when applied in this context. Two board-certified veterinarians trained in clinical coding independently evaluated MetaMap-extracted terms from 19 randomly-selected records. Disagreements were resolved via in-depth discussion and consensus. Evaluation of the NLP was done using a confusion matrix for terms detected in each record. The ground truth in this evaluation was considered to be the human experts; i.e., terms selected by MetaMap but not by reviewers were considered false positives, terms selected by reviewers but not by MetaMap were considered false negatives, and terms selected by both the reviewers and MetaMap were considered true positives. We assumed there were no true negatives. This process resulted in a weighted-average precision of 0.62, recall of 0.82, and F_1_ score of 0.71 for the CSU data, as compared to a previously reported weighted-average precision of 0.67, recall of 0.53, and F_1_ score of 0.58 for human clinical narratives. The aggregate results of this evaluation can be seen in S1 Table.

The entities recognized by MetaMap included all semantic types described in the website documentation (<https://metamap.nlm.nih.gov/Docs/SemanticTypes_2018AB.txt>). We did not restrict the search to any particular semantic type. Evaluation of the NLP was done using a confusion matrix for each record. For the first example, the confusion matrix would be as shown in S2 Table.

In our current design, it is not possible to know how many entities were missed by both the MetaMap tagger and the two board-certified veterinarians with clinical coding training. We assumed this number to be zero. Given this assumption, the evaluation of MetaMap in veterinary narratives is comparable to previously reported evaluations in human narratives.

After MetaMap selected terms and assigned concept-unique identifiers (CUI), we only retained the matched term. The order in which terms appear in the original text was also preserved. Finally, we constructed MetaMap-matched clinical narratives, where those terms assigned “False” by the ConText algorithm are added a “no_” prefix. All terms were converted to lowercase, special characters (not alphanumeric) were removed, and extraneous spaces were simplified to single underscores. When multiple matched CUIs existed, duplicates of the word were kept in the new clinical narrative. This process was conducted independently for each document before passed as input into the deep learning model. S3 Table shows an example of one free-text clinical narrative processed with MetaMap.
