# Supplementary figures and images for: FasTag: Automatic text classification of unstructured medical narratives
Source: PLoS One. 2020 Jun 22;15(6):e0234647. doi: 10.1371/journal.pone.0234647 (PMC7307763; doi:10.1371/journal.pone.0234647)

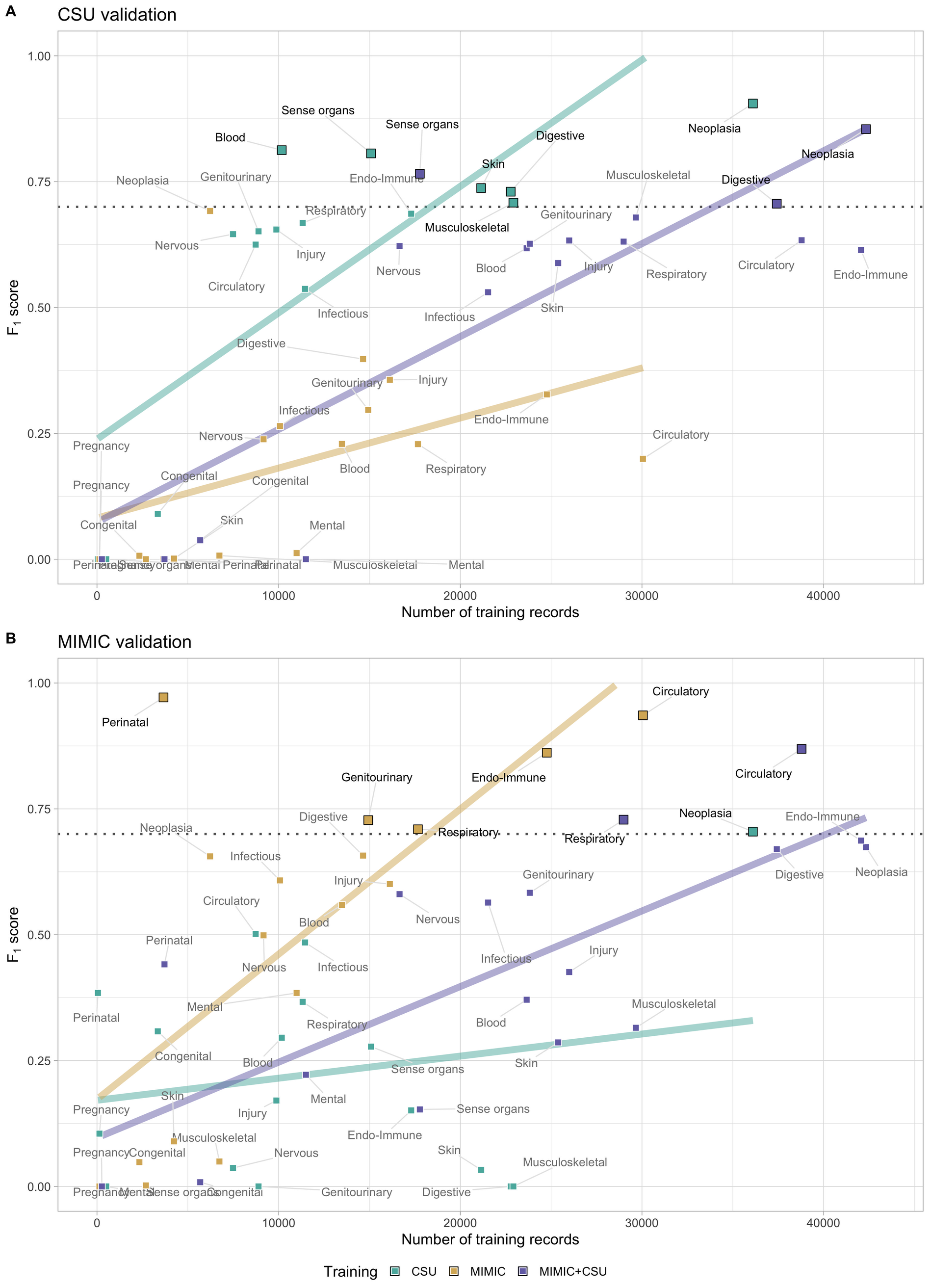

Supplement: S1 Fig — Training with CSU data (green), MIMIC data (yellow) or MIMIC+CSU (purple); validating on CSU data (Panel A) or MIMIC data (Panel B). The color of the category text is darkened (black) and the box made bigger if it surpasses the threshold of an F1 score of at least 0.70 (dotted horizontal line). (TIF) [file pone.0234647.s006.tif]
